# Chromodomain Helicase/ATPase DNA-Binding Protein 1-Like Gene (CHD1L) Expression and Implications for Invasion and Metastasis of Breast Cancer

**DOI:** 10.1371/journal.pone.0143030

**Published:** 2015-11-23

**Authors:** Qing-Jie Mu, Hong-Li Li, Yuan Yao, Shi-Chao Liu, Chong-Gao Yin, Xue-Zhen Ma

**Affiliations:** 1 Qing Dao University, Qingdao, PR China; 2 Clinical Department, Weifang Medical University, Weifang, PR China; 3 Medicine Research Center, Weifang Medical University, Weifang, 261053, PR China; 4 Affiliated Hospital of Qing Dao University, Qing dao, 260003, PR China; 5 Qing dao Central Hospital Qing Dao Tumor Hospital, the Second Affiliated hospital of Qing Dao University, Qing dao, 260042, PR China; 6 College of Nursing, Weifang Medical University, Weifang, 261053, PR China; INRS, CANADA

## Abstract

**Background:**

Chromodomain helicase/ATPase DNA-binding protein 1-like gene (*CHD1L*), also known as ALC1 (amplified in liver cancer 1 gene), is a new oncogene amplified in many solid tumors. Whether this gene plays a role in invasion and metastasis of breast cancer is unknown.

**Methods:**

Immunohistochemistry was performed to detect the expression of CHD1L in patients with invasive ductal carcinoma and normal mammary glands. Chemotaxis, wound healing, and Transwell invasion assays were also performed to examine cell migration and invasion. Western blot analysis was conducted to detect the expression of CHD1L, MMP-2, MMP-9, pAkt/Akt, pARK5/ARK5, and pmTOR/mTOR. Moreover, ELISA was carried out to detect the expression levels of MMP-2 and MMP-9. Nude mice xenograft model was used to detect the invasion and metastasis of breast cancer cell lines.

**Results:**

CHD1L overexpression was observed in 112 of 268 patients (41.8%). This overexpression was associated with lymph node metastasis (*P* = 0.008), tumor differentiation (*P* = 0.020), distant metastasis (*P* = 0.026), MMP-2 (*P* = 0.035), and MMP-9 expression (*P* = 0.022). In the cell experiment, reduction of CHD1L inhibited the invasion and metastasis of breast cancer cells by mediating MMP-2 and MMP-9 expression. CHD1L knockdown via siRNA suppressed EGF-induced pAkt, pARK5, and pmTOR. This knockdown inhibited the metastasis of breast cancer cells into the lungs of SCID mice.

**Conclusions:**

CHD1L promoted the invasion and metastasis of breast cancer cells via the PI3K/Akt/ARK5/mTOR/MMP signaling pathway. This study identified CHD1L as a potential anti-metastasis target for therapeutic intervention in breast cancer.

## Introduction

Breast cancer is the leading cause of cancer death in women worldwide, but it is not fatal in its early stages. The survival rate of this type of cancer falls from 90% for localized breast cancer to 20% for metastatic breast cancer [1]. Metastasis is the major cause of mortality among breast cancer patients. Metastatic breast cancer results from several sequential steps in which the tumor cells detach from the original tissue, intravasate into blood vessels, survive and travel along the circulation, extravasate to secondary organs, and proliferate [2]. The relation between numerous molecular markers, such as growth factors, cell adhesion proteins, and cell cycle regulators, and cancer metastasis has recently been investigated.

Chromodomain helicase/adenosine triphosphatase DNA-binding protein 1-like gene (CHD1L) is also known as amplified in liver cancer 1 gene (ALC1). This gene belongs to the sucrose nonfermenting 2 (SNF2)-like subfamily of the SNF2 family. SNF2 proteins play important roles in transcriptional regulation, DNA repair, and maintenance of chromosome integrity [3]. CHD1L is a recently identified oncogene localized at 1q21 in hepatocellular carcinoma (HCC) [4]. This gene can facilitate carcinogenesis mainly because of its epithelial–mesenchymal transition-inducing effects and anti-apoptosis in HCC [5,6]. CHD1L protein is overexpressed in human bladder, ovarian, and colorectal carcinomas and is a novel predictive biomarker for cancer patient survival [7–9]. However, the underlying molecular mechanism of CHD1L in promoting invasion and metastasis of breast cancer is unclear.

Previous studies proved that high MMP-2 and MMP-9 expression can promote breast cancer invasion [10]. PI3K/Akt/mTOR is hyperactive in more than 70% of breast tumors and is a crucial element within numerous complex signaling networks [11,12]. Activation of mTOR enhances the translation of certain mRNAs, including MMPs [13,14]. Moreover, Akt and mTOR are important downstream mediators of the effects of PI3K. AMP-activated protein kinase (AMPK)-related kinase 5 (ARK5; also known as NUAK1) is a serine/threonine kinase that belongs to the AMPK family [15]. ARK5 plays an important role in mediating cancer cell migration activity. Akt-dependent phosphorylation at Ser 600 can induce ARK5 activation [16]. ARK5 is an upstream AMPK regulator and can limit protein synthesis via inhibiting the mTORC1 signaling pathway [17].

This study shows that the correlation of CHD1L expression in breast tissues with the clinicopathological characteristics of patients and positive expression of CHD1L is associated with invasion and distant metastasis. CHD1L promoted invasion and metastasis of breast cancer cells. CHD1L played an important role in the PI3K/Akt/ARK5/mTOR/MMP signaling pathway *in vitro* and in an SCID mouse model. Thus, our study suggests that CHD1L could be a useful marker to predict tumor progression and a potential target for breast cancer therapy.

## Methods

### Patients and tissue specimens

Paraffin blocks from breast tissue specimens were obtained from the Affiliated Hospital of Weifang Medical University from 2006 to 2010, guided by a protocol approved by the Affiliated Hospital of Weifang Medical University-Institutional Review Board (AHWMU-IRB). Patients gave consent to the use of their tissues and provided written informed consent in this study. These tissues consisted of samples from 268 cases of invasive ductal carcinoma and 150 normal mammary glands. Clinical information of the patients is described in detail in Table 1.

**Table 1:** Association between CHD1L expression and clinical features of invasive ductal carcinoma patients.

Variables	CHD1L expression		*p* Value
	Positive expression	Negative expression	
**Age (years)**			
**≤50**	50	69	0.523
**≥51**	62	87	
**Tumor size (cm)**			
**≤5cm**	44	76	0.140
**>5cm**	68	80	
**Tumor grade**			
**I**	28	60	0.020
**II**	34	50	
**III**	50	46	
**Lymph node metastasis**			
**Yes**	60	65	0.008
**No**	52	91	
**Distant metastasis**			
**Yes**	66	72	0.026
**No**	46	84	
**ER**			
**Positive**	51	73	0.468
**Negative**	61	83	
**PR**			
**Positive**	55	77	0.485
**Negative**	57	79	
**CerbB2**			
**Positive**	53	69	0.465
**Negative**	59	87	
**MMP-2**			
**Positive**	62	66	0.035
**Negative**	50	90	
**MMP-9**			
**Positive**	64	67	0.022
**Negative**	48	89	

### Immunohistochemistry

To study altered protein expression in all human breast tissues, we utilize streptavidin-peroxidase assay according to the manufacturer’s instructions. The antibodies and the dilution factors were as follows: CHD1L (Abcam, 1:200), PR (Santa Cruz biotechnology, 1:200), ER (Santa Cruz biotechnology, 1:200), CerbB-2 (Santa Cruz biotechnology, 1:200). The degree of immunostaining of sections was reviewed and scored independently by two observers, based on both the proportion of positively stained tumor cells and the intensity of staining [18]. The cells at each intensity of staining were recorded on a scale of 0 = no staining, 1 = weak staining, 2 = moderate staining, and 3 = strong staining. The proportion of positively stained tumor cells was graded as follows: 0 = no positive tumor cells, 1<10% positive tumor cells, 2 = 10–50% positive tumor cells and 3>50% positive tumor cells). The staining index = staining intensity × proportion of positively stained tumor cells. We evaluated the expression level of CHD1L, MMP-2 and MMP-9 by staining index (scored as 0, 1, 2, 3, 4, 6, or 9) using this method. The staining index score was graded as negative (scored as 0–1) or positive (2–9) expression. The ER, PR and CerbB2 status of surgical specimens were determined by IHC. Positive staining for ER/PR was defined as nuclear staining in ≥1% of tumor cells. CerbB2 positivity was considered as CerbB2 3+ by IHC.

### Cell lines and generation of cell lines

All culture media and related reagents were purchased from HyClone (Logan, UT). The human breast cancer cell lines MDA-MB-231, T-47D, SK-BR-3 and MCF-7 were obtained from American Type Culture Collection (ATCC). The MDA-MB-231 cells were cultured in L-15 supplemented with 10% fetal bovine serum, at 37°C in a humidified atmosphere of 100% air. MCF-7 cells were cultured in MEM supplemented with 10% fetal bovine serum, at 37°C in a humidified atmosphere of 95% air and 5% CO_2_. T-47D and SK-BR-3 cells were cultured in DMEM supplemented with 10% fetal bovine serum, at 37°C in a humidified atmosphere of 95% air and 5% CO_2_.

Silencing of CHD1L was carried out using CHD1L-specific siRNA plasmid and control scrambled siRNA plasmid from Genechem Co. The sequences were No.1: 5’- TATTGGACATGCCACGAAA -3’, No.2: 5’-CAAGAGAAGGAGACTCATA -3’, No.3: 5’- ACAAACTCTTGCAGCCATT-3’, No.4: 5’- TGTCTTTAGCAACCAGCTA-3’ and TTCTCCGAACGTGTCACGT for controlled scrambled siRNA plasmid. Stably transfected cells were obtained by using 600 μg/mL puromycin. Surviving cells were assessed for CHD1L expression by Western blot.

CHD1L cDNA was cloned in the XhoI-KpnI sites of GV230. MCF-7 cells were transfected with GV230-CHD1L plasmid or GV230 vector using Lipofectamine 2000 according to the manufacturer’s instructions. Stable transfected cells were obtained by using 400 μg/mL G418. Single cell clones were isolated for clone expansion. Stable transfected cell clones were maintained and passaged in culture medium with 200 μg/mL G418.

### RNA extraction and real-time qRT-PCR

Total RNA was extracted from cells and tissues using TRIzol Reagent, and equal amounts of RNA were used for real-time qRT-PCR analysis according to the manufacturer’s instructions. β-actin was used as an internal control. Below are the specific primers used: CHD1L [19], sense, 5'-GGTGGAGTTGGCATGAACTT -3' and antisense 5'- CACTCAACTGGAGGTCAGCA -3'; β-actin, sense, 5’-TCCTGTGGCATCCACGAAACT-3’ and antisense 5’- GAAGCATTTGCGGTGGACGAT-3’. The 2^-ΔΔCT^ method was used to calculate the relative abundance of RNA for each gene compared with β-actin expression. Each reaction was performed in triplicate.

### Chemotaxis assay

Chemotaxis assays were performed by using transwell chamber. Briefly, cells suspended in the binding medium were added to the upper chambers and EGF was loaded into the lower chemotaxis chamber. The polycarbonate filter was pretreated with 10 μg/ml fibronectin overnight, dried in air, and inserted between the upper and lower chambers. Then, the chamber was incubated at 37°C in 5% CO_2_ for 3 h. The filter membrane was then rinsed, fixed, and stained. The numbers of migrating cells were counted at 400× in three separate fields by light microscope.

### Western blot

Western blot analyses were performed using the standard method with antibody. Antibodies used were: anti-CHD1L (Abcam, 1:500), MMP-2 (Santa Cruz biotechnology, 1:1000), MMP-9 (Santa Cruz biotechnology, 1:1000), p-Akt (Cell Signaling Technology, 1:1000), Akt (Cell Signaling Technology, 1:1000), p-ARK5 (Cell Signaling Technology, 1:1000), ARK5 (Cell Signaling Technology, 1:1000) antibody. All experiments were repeated at least 3 times. The densitometry data were analyzed using Image J software.

### Wound healing assay

All cells were seeded on a 6-well plate and incubated overnight. After making a straight scratch by using a 10 μL pipette tip, cells were incubated in a minimum medium containing 0.1% BSA at a 37°C in 5% CO_2_ humidified incubator. The wells were washed twice with PBS and fresh medium supplemented with or without 10 ng/mL EGF was added. To assess cell migration into wound, we took photographs at 0h, 12h and 24h.

### Transwell invasion assay

In order to detect the invasion of breast cancer cells, an invasion assay using 24-well Matrigel invasion chambers was performed. Briefly, 1.5 mg/mL of Matrigel inserts were prepared according to protocol. The cells were added in serum-free medium and incubated at 37°C for 30 min. About 300 μL of binding medium including L-15 or MEM, 0.1% BSA, and 25 mM HEPES with 10 ng/mL of EGF was added to the lower well. After 24 h of incubation, the cells were washed twice with PBS and stained with Giemsa for 1 h and photographed under a microscope.

### Enzyme-linked immunosorbent assay (ELISA)

An ELISA kit was used to detect the MMP-2 and MMP-9 expression levels in the culture medium of all cells with or without 10 ng/mL EGF. The cells were incubated for a further 24 h until about 80% confluence was attained. The medium was then harvested and filtered for the measurement of MMP-2 and MMP-9. Each experiment was repeated three times.

### Xenograft model in nude mice

Six-week-old NOD/LtSz-*scid/scid* mice were purchased from Wei Tong Li Hua Experimental Animal Company. Twenty SCID mice were divided randomly into two groups. ScrMDA231 or SiCHD1L/MDA231 cells were injected into their mammary fat pads. Body weight and tumor volume were measured once a week and the tumor volume was calculated according to the formula: V = (L × W^2^) × 0.5, where “L” and “W” were the length and width of a xenograft. After 8 weeks, the mice were killed. All surgeries were performed under isofluorane anesthesia, and all efforts were made to minimize suffering. The lung tissues were surgically removed under euthanization, and then were fixed with formalin and embedded in paraffin to examine metastasis. Serial sections and H&E staining were performed to detect lung micrometastasis. All the animal protocols were approved by the Institutional Animal Care and Use Committee of Weifang Medical University. All the animal experiments were monitored by the Department of Laboratory Animal Resources of Weifang Medical University. All sections of this report adhere to the ARRIVE Guidelines for reporting animal research [20]. A completed ARRIVE guidelines checklist is included in S1 ARRIVE Checklist.

### Statistical analysis

All data are presented as mean ± SD. Statistical significance for comparisons between groups was determined using Student’s paired two-tailed t-test or ANOVA. Chi-square test was used to analyze the relationship between CHD1L protein expression and the clinicopathologic characteristics of breast cancer. Survival curve was plotted using the Kaplan–Meier method and was compared using the log-rank test. All the results were generated from three independent experiments. *P* < 0.05 was considered statistically significant.

## Results

### CHD1L is specifically upregulated in human breast cancer cell lines and tissues

**Fig 1:**
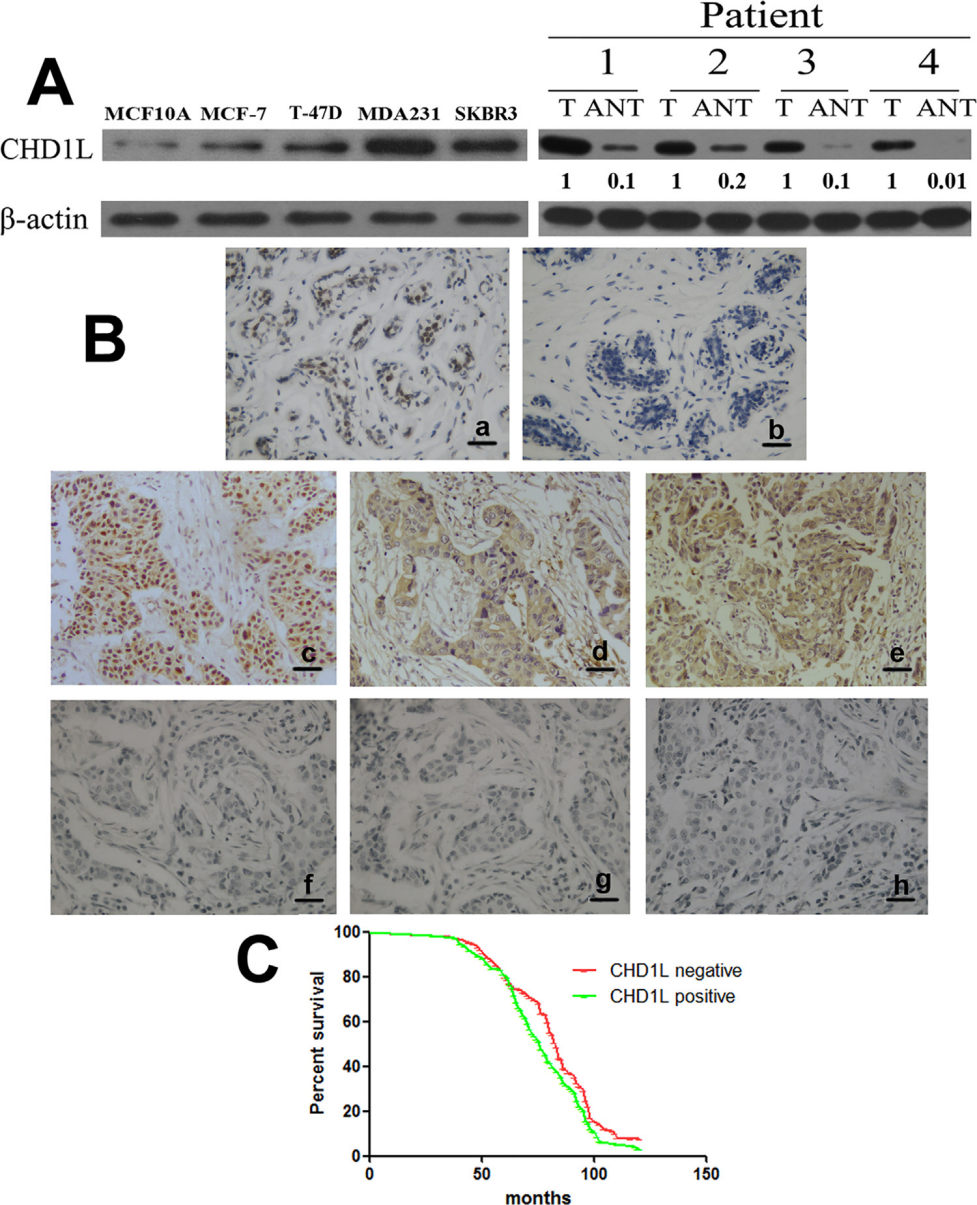
CHD1L is specifically upregulated in human breast cancer cell lines and tissues. (A) Left, Expression of CHD1L protein in cultured human normal mammary epithelial cell line (MCF10A) and various breast cancer cell lines (MDA-MB-231, T-47D, SK-BR-3, and MCF-7). Right, Expression of CHD1L protein in paired breast cancer tissues (T) and adjacent non-tumor tissues (ANT), with each pair obtained from the same patient. β-Actin was used as a loading control. Quantification of relative protein levels is shown below the blots. Results are representative of at least three repeated experiments. (B) Positive expression of CHD1L protein in normal mammary glands (a), negative expression of CHD1L protein in normal mammary glands (b), positive expression of CHD1L protein in carcinoma with lymph node metastasis (c), positive expression of MMP-2 protein in carcinoma with positive expression of CHD1L (d), positive expression of MMP-9 protein in carcinoma with positive expression of CHD1L (e), negative expression of CHD1L protein in carcinoma without lymph node metastasis (f), negative expression of MMP-2 protein in carcinoma with negative expression of CHD1L (g), and negative expression of MMP-9 protein in carcinoma with negative expression of CHD1L (h) were examined using immunohistochemical staining. Scale bar: 20 μm. (C) Kaplan–Meier analysis was performed between the CHD1L level and overall survival of breast cancer patients with positive (n = 112) and low (n = 156) CHD1L expression.

We initially detected the expression of CHD1L in different cell lines through real-time PCR and Western blot analyses. The expression of CHD1L protein and mRNA differed in MCF10A (human normal mammary epithelial cell line) and the two breast cancer cell lines (MCF-7, T-47D, SK-BR-3, and MDA-MB-231). MDA-MB-231 cells expressed higher CHD1L, whereas MCF-7 cells expressed lower CHD1L (Fig 1A, left; S1 Fig). Furthermore, comparative evaluation of paired adjacent non-tumor tissues (ANT) and breast cancer tumor, with each pair obtained from the same patient, was conducted using Western blot. The level of CHD1L protein was higher in breast cancer tissues than that of their corresponding ANT tissues (Fig 1A, right).

CHD1L expression in breast tissues was determined using immunohistochemical analysis. CHD1L was expressed in 112 of 268 invasive ductal carcinoma tissues (41.8%) and 32 of 150 normal mammary glands (21.3%) (Fig 1B). The staining intensity of all the normal breast tissue was 1+ or negative, and the percentage of positive cells was all less than 50% (Fig 1B). The CHD1L expression in normal mammary glands was significantly lower than that in invasive ductal carcinoma (*P* < 0.001).

The correlations between the expression of CHD1L and the clinicopathological parameters of invasive ductal carcinoma were analyzed. Table 1 shows that the CHD1L expression in breast cancer was significantly associated with lymph node metastasis (*P* = 0.008), tumor grade (*P* = 0.020), distant metastasis (*P* = 0.026), MMP-2 (*P* = 0.035), and MMP-9 expression (*P* = 0.022) (Fig 1B). Significant association was not found between CHD1L expression and tumor size, age, and hormonal status (Table 1).

Kaplan–Meier analysis was performed using the log-rank test. Patients with negative CHD1L expression in their lung tumors had longer median survival time of 83 months compared with those with positive CHD1L expression, whose median survival time was 76 months (*P* = 0.031) (Fig 1C).

### CHD1L promotes chemotaxis of breast cancer cells

**Fig 2:**
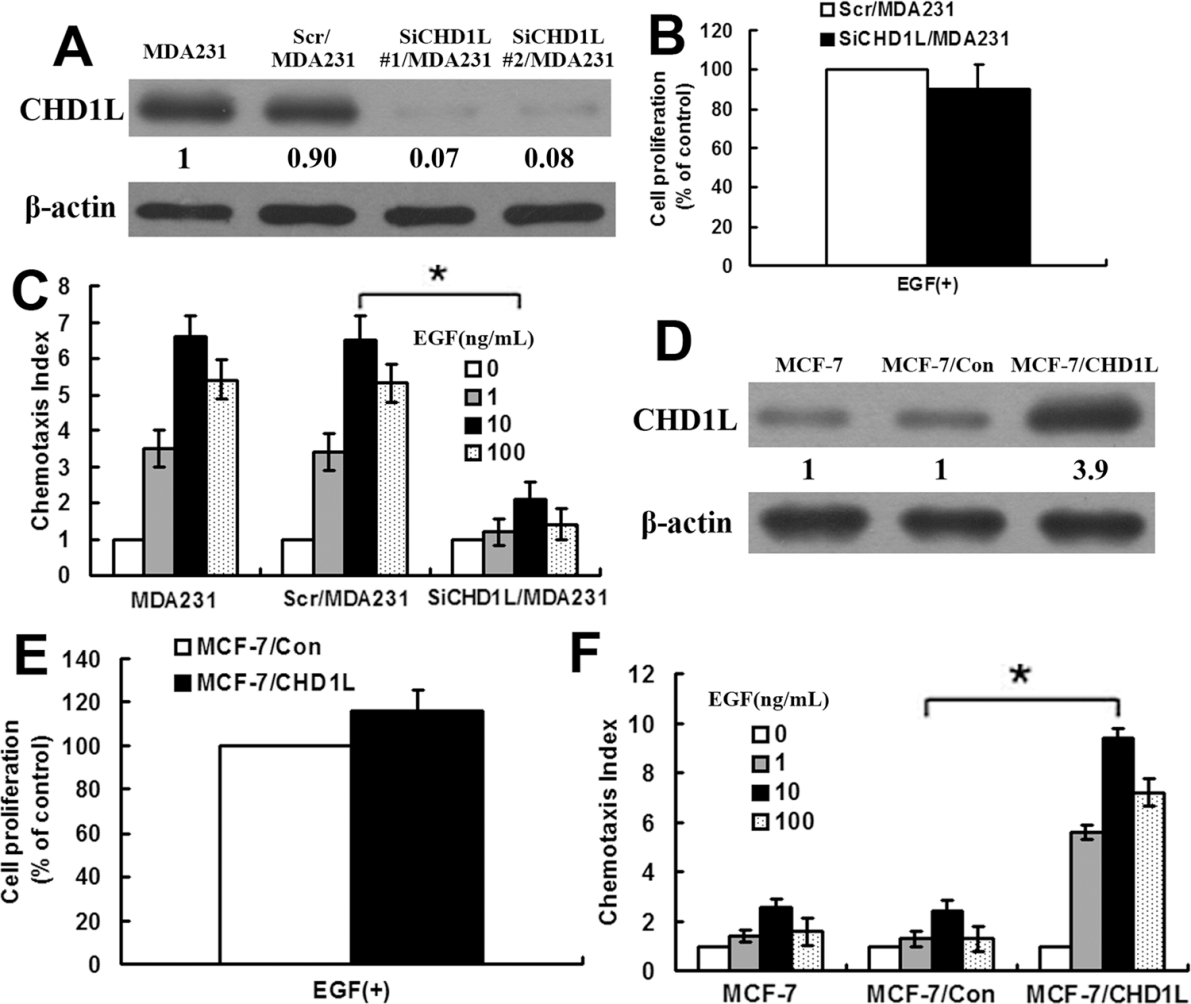
CHD1L promotes chemotaxis of breast cancer cells. (A) Expression of CHD1L protein in MDA-MB-231 cells (MDA231), MDA-MB-231 cells transfected with control scrambled siRNA (Scr/MDA231), and MDA-MB-231 cells transfected with two sets of stable siRNA-targeting CHD1L (SiCHD1L#1/MDA231 and SiCHD1L#2/MDA231) was detected using Western blot. β-Actin was used as a loading control. Quantification of relative protein levels on three different Western blot analyses is shown below the blots. (B) Comparison of cell proliferation in Scr/MDA231 and SiCHD1L/MDA231 (SiCHD1L#1/MDA231). Each data point was an average of triplicate assays. (C) Cell chemotaxis ability was assessed using chemotaxia assay in MDA-MB-231, Scr/MDA231, and SiCHD1L/MDA231 (SiCHD1L#1/MDA231) with EGF stimulation. Columns, mean of triplicate measurements. Bars, standard deviation. *, *P* < 0.05 (Student’s t test, versus MDA-MB-231 or Scr/MDA231). (D) Expression of CHD1L protein in MCF-7 cells, MCF-7/Con cells (transfected with GV230 vector), and MCF-7/CHD1L cells (stably transfected with GV230-CHD1L, stable clone 4) was detected using Western blot. β-Actin was used as a loading control. Quantification of relative protein levels on three different Western blot analyses is shown below the blots. (E). Comparison of cell proliferation in MCF-7/Con and MCF-7/CHD1L. Each data point was an average of triplicate assays. (F) Comparison of chemotactic abilities with EGF stimulation in MCF-7, MCF-7/Con, and MCF-7/CHD1L. The data collected in this set of figures are representative of at least three independent experiments. Columns, mean of triplicate measurements. Bars, standard deviation. *, *P* < 0.05 (Student’s t test, versus MCF-7 or MCF-7/Con).

To determine the effect of CHD1L on the migration of MDA-MB-231 cells, we inhibited CHD1L expression in MDA-MB-231 cell line through siRNA experiments. Four siRNAs targeting CHD1L were detected for their efficiency of CHD1L gene silencing, and siRNA duplex no. 1 effectively lowered CHD1L protein expression. The cell lines that stably downregulated CHD1L protein were selected through puromycin. Two representative clones (clones 1 and 2) were used in the analysis. These clones had similar phenotypes; we selected clone 1, designated as SiCHD1L/MDA231 cells, as representative. We transfected an siRNA construct that contains a scrambled sequence into MDA-MB-231 cells, designated as Scr/MDA231 cells, as control. The efficacy of CHD1L knockdown was confirmed via real-time PCR and Western blot analyses in SiCHD1L/MDA231 cell lines (Fig 2A; S2A Fig). The proliferation rates of Scr/MDA231 and SiCHD1L/MDA231 cells were examined *in vitro*. These cells exhibited unchanged proliferation rates after 12 h of EGF stimulation (Fig 2B). The parental MDA231 and Scr/MDA231 cells showed similar chemotaxis ability; however, the chemotaxis ability of SiCHD1L/MDA231 cells was evidently decreased compared with that of Scr/MDA231 cells (Fig 2C).

The full-length cDNA of CHD1L gene was cloned into GV230 and stably transfected into MCF-7 cell clones. Western blot detected the expression of CHD1L protein in transfected cells. Thus, the results from clone 2, designated as MCF-7/CHD1L cells, were presented as representative. MCF-7 cells were transfected with a GV230 vector, which were designated as MCF-7/Con. The protein and mRNA expression of stably transfected CHD1L clones are illustrated in Fig 2D and S2B Fig. Cell proliferation rate was also examined in both MCF-7/Con and MCF-7/CHD1L cells *in vitro*. These cells exhibited unchanged proliferation rates after 12 h of EGF stimulation (Fig 2E). The chemotaxis ability of MCF-7/CHD1L cells was evidently increased compared with that of MCF-7/Con cells (Fig 2F). These results indicate that CHD1L played an important role in the EGF-induced chemotaxis of breast cancer cells.

### CHD1L promotes invasion of breast cancer cells

**Fig 3:**
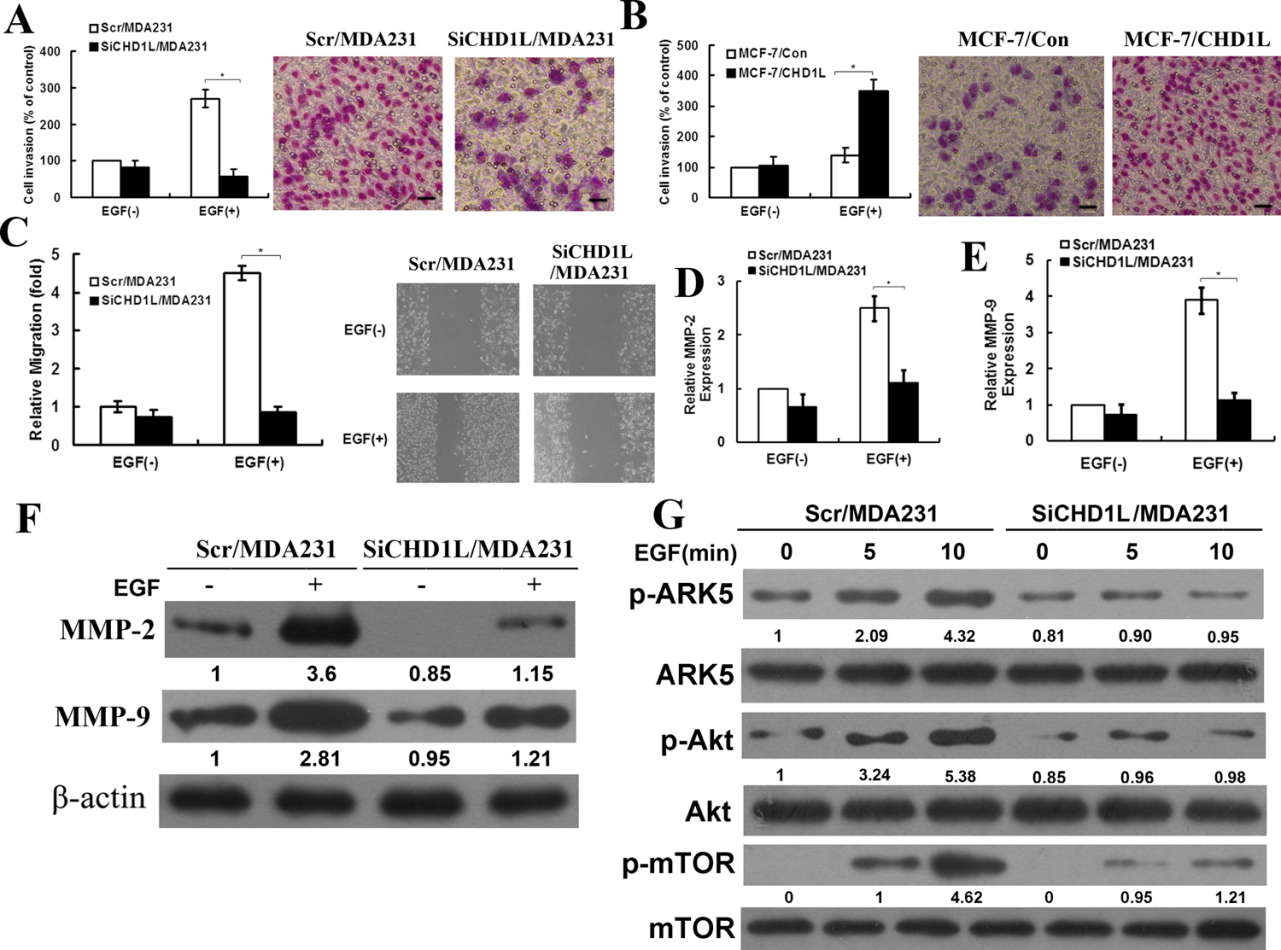
CHD1L promotes invasion of breast cancer cells. (A) Invasion of breast cancer cell line MDA-MB-231 with EGF stimulation was analyzed. Left, Quantification of penetrated cells was analyzed. Bars, standard deviation. *, *P* < 0.05 (Student’s t test, versus Scr/MDA231 with EGF stimulation). Right, Images were taken at a magnification. Scale bar: 20 μm. (B) Invasion of breast cancer cell line MCF-7 with EGF stimulation was analyzed. Left, Quantification of penetrated cells was analyzed. Bars, standard deviation, *, *P* < 0.05 (Student’s t test, versus MCF-7/Con with EGF stimulation). Right, Images were taken at a magnification. Scale bar: 20 μm. (C) Cell migration was assessed using the wound healing assay. Left, Cell motility rates of SiCHD1L/MDA231 and Scr/MDA231. Bars, standard deviation. *, *P* < 0.05 (Student’s t test, versus Scr/MDA231 with EGF stimulation). EGF, 10 ng/mL. Right, Images were taken at 0 and 24 h. (D) MMP-2 level in culture media of SiCHD1L/MDA231 and Scr/MDA231 cells was detected using ELISA. EGF, 10 ng/mL. Bar, standard deviation. *, *P* < 0.05 (Student’s t test, versus Scr/MDA231 with EGF stimulation). (E) MMP-9 level in culture media of SiCHD1L/MDA231 and Scr/MDA231 cells was assessed using ELISA assay, EGF, 10 ng/mL. Bars, standard deviation. *, *P* < 0.05 (Student’s t test, versus Scr/MDA231 with EGF stimulation). (F) MMP-2 and MMP-9 proteins were extracted in Scr/MDA231 and SiCHD1L/MDA231 cells with or without EGF stimulation, and Western blot was performed using an antibody to MMP-2 and MMP-9. β-Actin was used as a loading control. (G) Protein levels of pAkt, Akt, pARK5, ARK5, pmTOR, and mTOR were detected using Western blot. Each result is representative of at least three independent experiments. Akt was used as a loading control. Quantification of relative protein levels on three different Western blot analyses is shown below the blots.

Breast cancer cell migration is essential in breast tumor invasion. Thus, we hypothesize that CHD1L plays a crucial role in breast cancer cell invasion. Matrigel invasion and wound healing assays were performed to determine the role of CHD1L in Scr/MDA231 and SiCHD1L/MDA231 cell invasion. EGF as a chemoattractant stimulated cell penetration through Matrigel. Compared with the Scr/MDA231 cells, the SiCHD1L/MDA231 cells showed dramatically reduced invasive ability (Fig 3A). The invasiveness of the MCF-7 cells also significantly increased after CHD1L upregulation (Fig 3B). Wound healing assay results showed that when monolayer cells were scratched, the cell motility rate clearly decreased in the SiCHD1L/MDA231 cells with EGF stimulation (Fig 3C).

MMP-2 and MMP-9 activities were assessed using ELISA with culture media of SiCHD1L/MDA231 and Scr/MDA231 cells. In the absence of EGF, significant changes in MMP-2 and MMP-9 levels were not observed in SiCHD1L/MDA231 cells. However, EGF treatment led to reduced MMP-2 and MMP-9 levels in SiCHD1L/MDA231 cells compared with Scr/MDA231 cells (Fig 3D and 3E). Furthermore, Western blot results also showed that the expression of MMP-2 and MMP-9 protein was significantly reduced after CHD1L downregulation with EGF stimulation (Fig 3F). This result is consistent with the ELISA data and strongly suggests that CHD1L downregulation is significantly involved in the reduced expression of MMP-2 and MMP-9 in breast cancer cell lines. We also detected the expression of MMP-2 and MMP-9 in MCF-7/con and MCF-7/CHD1L cells. The results are shown in S3 Fig. Therefore, MMP-2 and MMP-9 are key factors in CHD1L that promote invasiveness of breast cancer cells.

The PI3K/Akt/mammalian target of rapamycin (mTOR) pathway can activate multiple oncogenic programs and is essential in regulating breast cancer cell growth. Thus, we assessed the phosphorylation of Akt, ARK5, and mTOR to investigate the activation of the PI3K/Akt/ARK5/mTOR pathway. The pAkt (phosphorylation of Akt), pARK5 (phosphorylation of ARK5), and pmTOR (phosphorylation of mTOR) were significantly reduced in SiCHD1L/MDA231 cells compared with those in the Scr/MDA231 cells with EGF stimulation (Fig 3G). These data suggest that CHD1L-mediated signaling activates Akt, ARK5, and mTOR and promotes breast cancer cell invasion through the Akt–ARK5–mTOR pathway.

### CHD1L promotes lung colonization of breast cancer *in vivo*

**Fig 4:**
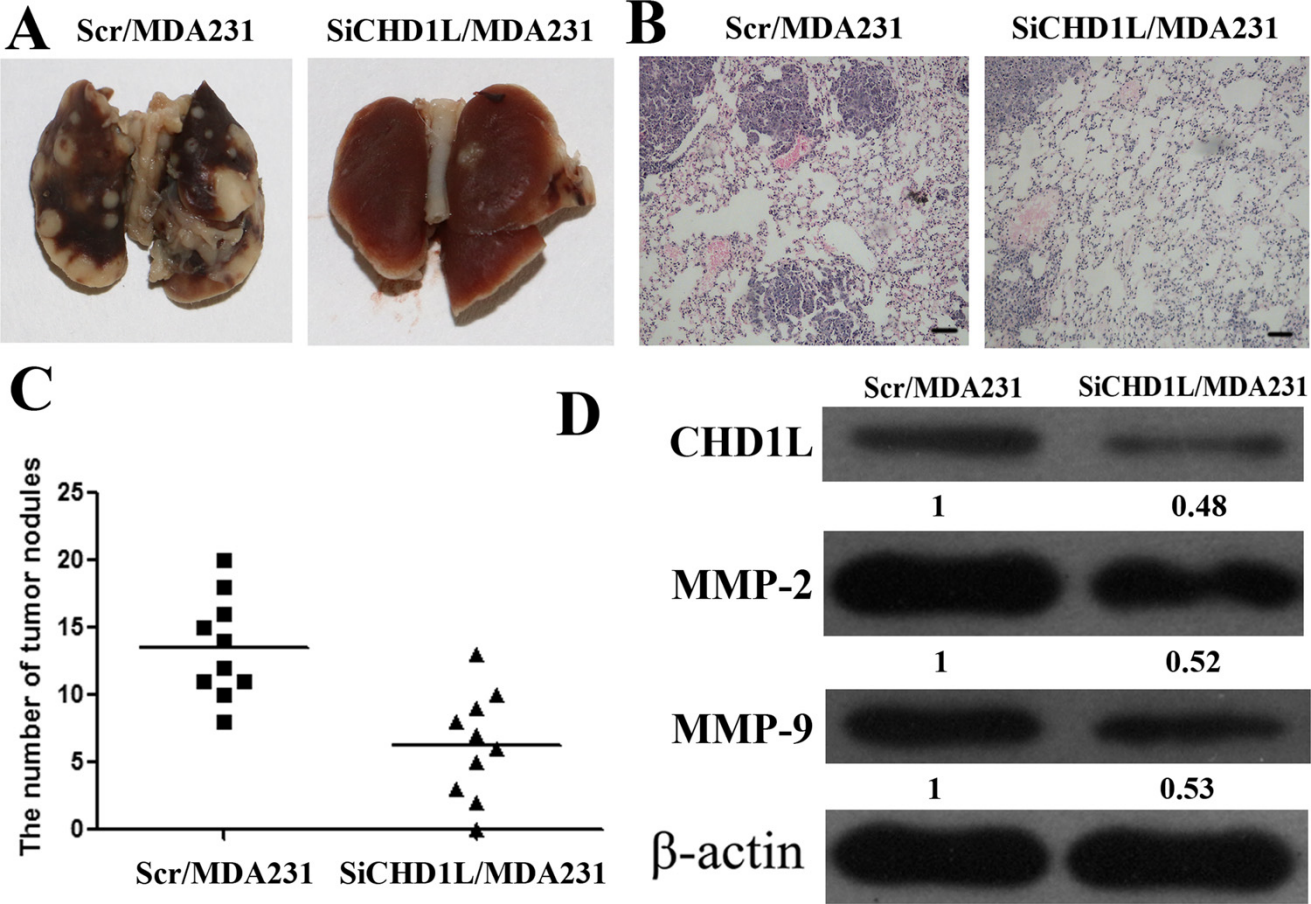
CHD1L promotes lung colonization of human breast cancer *in vivo*. (A) Comparison of tumor cell colonies in mouse lungs between groups. (B) Tumor foci in mouse lungs were visualized using H&E staining. Scale bar: 50 μm. (C) Lung metastatic nodules were counted (*n* = 10). (D) Expression of CHD1L, MMP-2, and MMP-9 was detected in mice xenograft tumors by Western blot analysis. β-Actin was used as a loading control. Quantification of relative protein levels on three different Western blot analyses is shown below the blots.

The metastatic properties of breast cancer cells *in vivo* were analyzed using a xenograft transplantation model in NOD/LtSz-*scid/scid* mice. Primary tumor volume was not significantly associated with Scr/MDA231 and SiCHD1L/MDA231. Tumor cell colonies in mouse lungs were examined through H&E staining. As shown in Fig 4A–4C, the number of metastatic tumor nodules decreased in the lungs of mice injected with SiCHD1L/MDA231. Simultaneously, the MMP-2 and MMP-9 protein expression of the tumor xenograft were downregulated in mice injected with SiCHD1L/MDA231 cells (Fig 4D). These findings are consistent with the *in vitro* results and indicate that CHD1L can promote the invasion and metastasis of breast cancer by promoting the PI3K/Akt/ARK5/mTOR/MMP signaling pathway.

## Discussion

*CHD1L* is a frequently amplified region in HCC and a recently identified oncogene localized at 1q21. CHD1L can contribute to tumor cell migration, invasion, and metastasis by increasing cell motility and inducing filopodia formation and EMT via ARHGEF9-mediated Cdc42 activation in HCC [6]. In this study, CHD1L was evidently upregulated in breast cancer tissues. This result indicates a significant correlation between CHD1L expression and lymph node metastasis of breast cancer. CHD1L can promote invasion and metastasis of breast cancer cells. CHD1L played an important role in the PI3K/Akt/ARK5/mTOR/MMP signaling pathway. In animal experiments, downregulation of CHD1L inhibited the metastasis of breast cancer cells toward the SCID mice lung. The results demonstrated the potential function of CHD1L in promoting breast cancer metastasis. These data indicate that CHD1L detection can provide useful information about CHD1L as a potential anti-metastasis target for therapeutic intervention in breast cancer.

Polarized cell migration is highly associated with the invasion and metastasis of tumors. This process is closely regulated and occurs during tissue development, wound healing, and chemotaxis [2]. In this study, the overexpression of CHD1L in the chemotaxis and wound healing assays highly promoted the migration ability of MCF-7 cells. This result suggests that CHD1L is an essential factor in breast cancer cell migration. MMPs play an important role in tumor invasion because these are key factors in the degradation of extracellular matrix and basement membranes [21]. MMP-2 and MMP-9 are complex members of the MMP family and demonstrate upregulated expression in breast cancer [22]. Previous studies showed the involvement of MMPs in Akt signaling in various cancer cell types [23,24]. The results of the present study show that EGF stimulation resulted in reduced MMP-2 and MMP-9 expression in CHD1L-downregulated cells compared with MDA231/con cells. Activation of the PI3K/Akt/mTOR signaling pathway in breast cancer could be as frequent as 70%. Several studies have suggested that the activation of PI3K/Akt/mTOR is associated with aggressive features, such as high histologic grade and poor clinical outcome [25]. Akt-dependent phosphorylation at Ser 600 can induce ARK5 activation. The phosphorylation of Akt, ARK5, and mTOR was significantly reduced in CHD1L-downregulating cells with EGF stimulation. This finding further indicates that a possible molecular mechanism underlying CHD1L participation in breast cancer invasion and metastasis involves the regulation of the PI3K/Akt/ARK5/mTOR signaling pathway.

CHD1L can promote the metastasis of human breast cancer cells in the lungs of SCID mice. This result suggests the importance of CHD1L in the metastasis of human breast cancer cells *in vivo*.

However, CHD1L affects the invasion and metastasis of breast cancer via several signal pathways both *in vitro* and *in vivo*. Further studies are warranted to demonstrate the function of CHD1L in many breast cancer cell lines and another signal pathway that affects the invasion and metastasis of breast cancer.

In summary, we showed that CHD1L levels were higher in breast cancer cells and that CHD1L promoted breast cancer cell migration, invasion, and metastasis. We also demonstrated the molecular mechanism by which CHD1L mediates cell migration, invasion, and metastasis. The findings in this study present a novel mechanism for EGF-induced invasion and metastasis and have important implications for the development of effective breast cancer treatment strategies.

## Supporting Information

S1 ARRIVE Checklist Completed ‘‘The ARRIVE Guidelines Checklist” for reporting animal data in this manuscript. (PDF)

S1 Fig Expression of CHD1L mRNA in cultured human normal mammary epithelial cell line (MCF10A) and various breast cancer cell lines (MDA-MB-231, T-47D, SK-BR-3, and MCF-7). (TIF)

S2 Fig (A) Expression of CHD1L mRNA in MDA-MB-231 cells (MDA231), MDA-MB-231 cells transfected with control scrambled siRNA (Scr/MDA231), and MDA-MB-231 cells transfected with two sets of stable siRNA-targeting CHD1L (SiCHD1L#1/MDA231 and SiCHD1L#2/MDA231) was detected using qRT-PCR. *, *P* < 0.05 (Student’s t test, versus Scr/MDA231 or MDA231). (B) Expression of CHD1L mRNA in MCF-7 cells, MCF-7/Con cells (transfected with GV230 vector), and MCF-7/CHD1L cells (stably transfected with GV230-CHD1L, stable clone 4) was detected using qRT-PCR. *, *P* < 0.05 (Student’s t test, versus MCF-7 or MCF-7/con). (TIF)

S3 Fig (A) MMP-2 level in culture media of MCF-7/con and MCF-7/CHD1L cells was detected using ELISA. EGF, 10 ng/mL. Bar, standard deviation, *, *P* < 0.05 (Student’s t test, versus MCF-7/con with EGF stimulation). (B) MMP-9 level in culture media of MCF-7/con and MCF-7/CHD1L cells was assessed using ELISA assay. EGF, 10 ng/mL. Bars, standard deviation. *, *P* < 0.05 (Student’s t test, versus MCF-7/con with EGF stimulation). (C) MMP-2 and MMP-9 proteins were extracted in MCF-7/con and MCF-7/CHD1L cells with or without EGF stimulation. Western blot was performed using an antibody to MMP-2 and MMP-9. β-Actin was used as a loading control. (TIF)
