# Supplementary figures and images for: Chromodomain Helicase/ATPase DNA-Binding Protein 1-Like Gene (CHD1L) Expression and Implications for Invasion and Metastasis of Breast Cancer
Source: PLoS One. 2015 Nov 23;10(11):e0143030. doi: 10.1371/journal.pone.0143030 (PMC4657932; doi:10.1371/journal.pone.0143030)

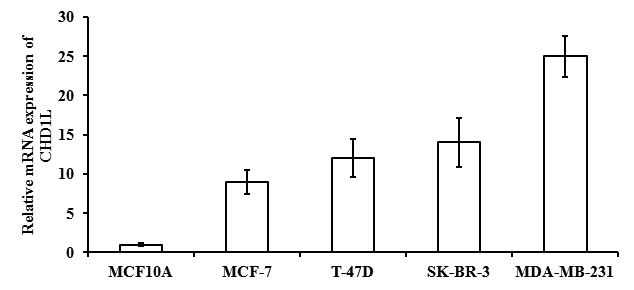

Supplement: S1 Fig — (TIF) [file pone.0143030.s002.tif]

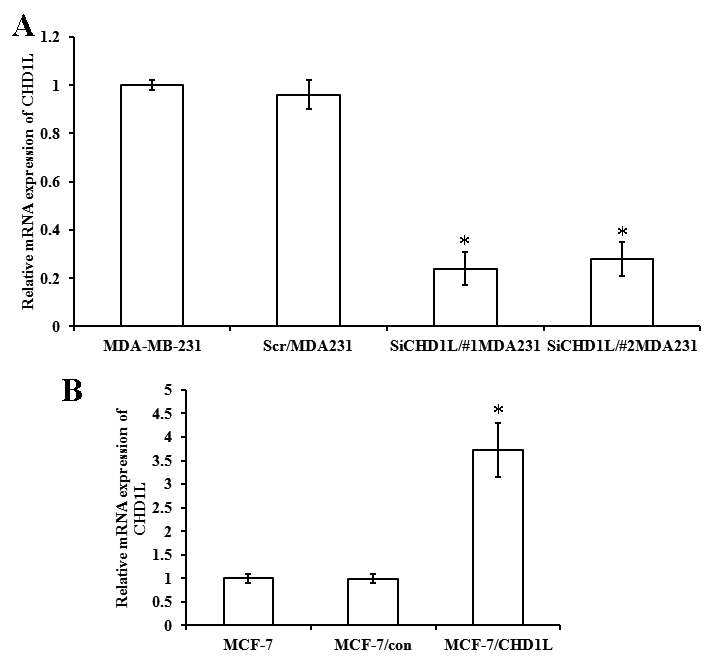

Supplement: S2 Fig — (TIF) [file pone.0143030.s003.tif]

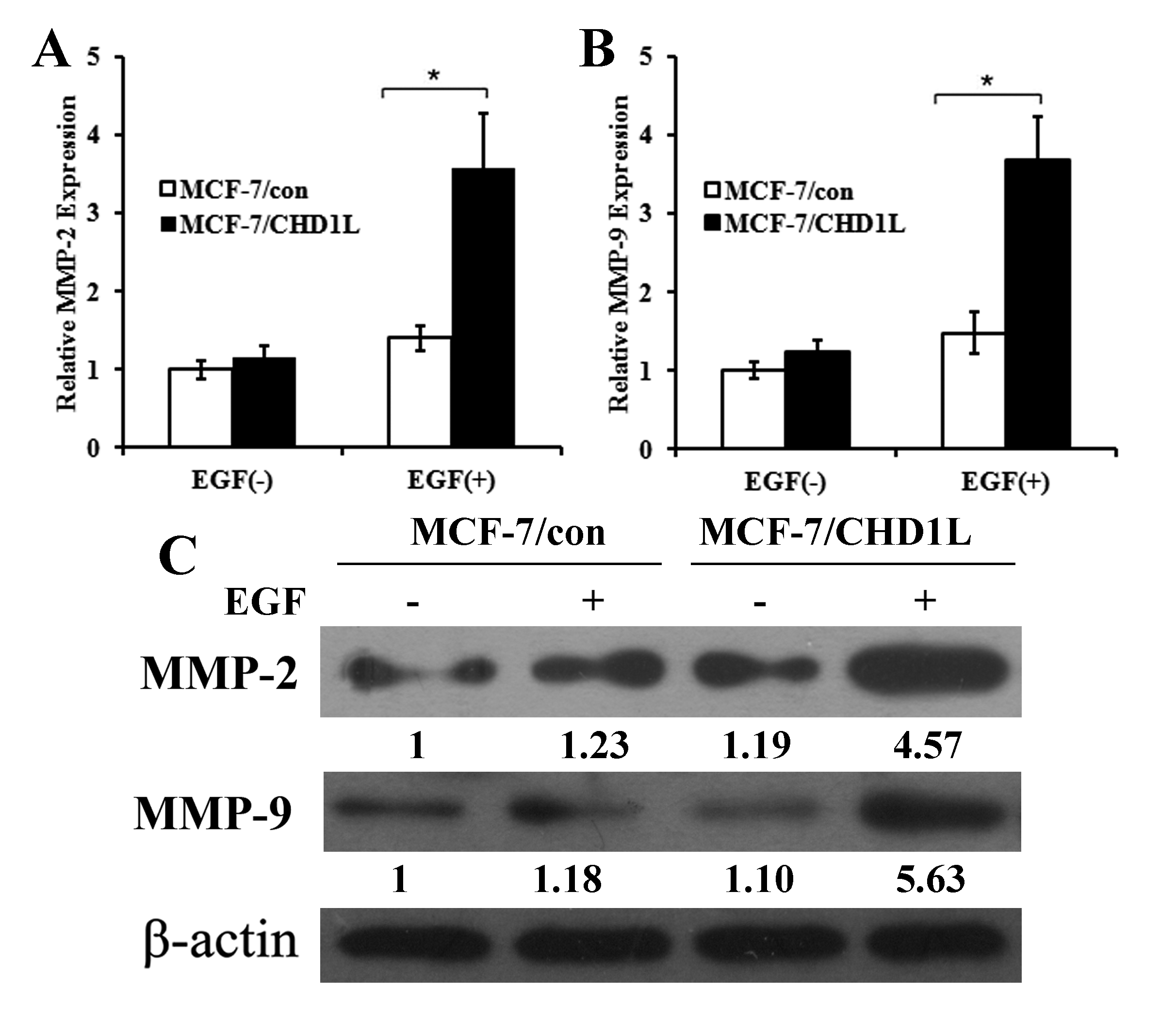

Supplement: S3 Fig — (TIF) [file pone.0143030.s004.tif]
